# The Use of Gamma-H2AX as a Biodosimeter for Total-Body Radiation Exposure in Non-Human Primates

**DOI:** 10.1371/journal.pone.0015544

**Published:** 2010-11-23

**Authors:** Christophe E. Redon, Asako J. Nakamura, Ksenia Gouliaeva, Arifur Rahman, William F. Blakely, William M. Bonner

**Affiliations:** 1 Laboratory of Molecular Pharmacology, Center for Cancer Research, National Cancer Institute, National Institutes of Health, Bethesda, Maryland, United States of America; 2 Armed Forces Radiobiology Research Institute, Uniformed Services University, Bethesda, Maryland, United States of America; National Cancer Institute, United States of America

## Abstract

**Background:**

There is a crucial shortage of methods capable of determining the extent of accidental exposures of human beings to ionizing radiation. However, knowledge of individual exposures is essential for early triage during radiological incidents to provide optimum possible life-sparing medical procedures to each person.

**Methods and Findings:**

We evaluated immunocytofluorescence-based quantitation of γ-H2AX foci as a biodosimeter of total-body radiation exposure (^60^Co γ-rays) in a rhesus macaque (*Macaca mulatta*) model. Peripheral blood lymphocytes and plucked hairs were collected from 4 cohorts of macaques receiving total body irradiation doses ranging from 1 Gy to 8.5 Gy. Each cohort consisted of 6 experimental and 2 control animals. Numbers of residual γ-H2AX foci were proportional to initial irradiation doses and statistically significant responses were obtained until 1 day after 1 Gy, 4 days after 3.5 and 6.5 Gy, and 14 days after 8.5 Gy in lymphocytes and until 1 day after 1 Gy, at least 2 days after 3.5 and 6.5 Gy, and 9 days after 8.5 Gy in plucked hairs.

**Conclusion:**

These findings indicate that quantitation of γ-H2AX foci may make a robust biodosimeter for analyzing total-body exposure to ionizing radiation in humans. This tool would help clinicians prescribe appropriate types of medical intervention for optimal individual outcome. These results also demonstrate that the use of a high throughput γ-H2AX biodosimeter would be useful for days post-exposure in applications like large-scale radiological events or radiation therapy. In addition, this study validates a possibility to use plucked hair in future clinical trials investigating genotoxic effects of drugs and radiation treatments.

## Introduction

In addition to routine therapeutic radiation exposures, substantial individual exposure to ionizing radiation may result from radiological accidents and other incidents [1], [2]. One major result from radiation exposure is the formation of DNA double strand breaks (DSBs), considered one of the most dangerous lesions for the induction of genomic alterations and cancer at low levels and substantial morbidity at higher levels.

The presence of DSBs can be determined with a sensitive quantitative assay based on the detection of phosphorylated H2AX (gamma-H2AX or γ-H2AX) foci at each break site, foci which can be visualized utilizing immunocyto- and immunohistochemistry techniques [3], [4]. Various human materials including lymphocytes, oral cells and skin biopsies have been used for the detection of γ-H2AX foci produced by ionizing radiation (IR) (reviewed in [5]). Lymphocytes from individuals subjected to low doses of IR from radiological diagnostic or therapeutic treatments [6], [7], [8], [9] and from humans traveling in space [10] were found to exhibit elevated levels of γ-H2AX foci and biodosimetry applications for the immunocytofluorescence γ-H2AX foci assay has been advocated ([11]; reviewed in [5] and [12]). Several groups are also actively engaged to automate various components of this assay [13], [14], [15].

However, there have been few systematic studies to characterize the attributes and limitations of this assay. While there are several studies describing γ-H2AX foci induction and loss in lymphocytes following exposure to non-life threatening doses and/or during procedures involving partial body irradiation (CT scan, angioplasty, radiotherapy, etc.) [6], [7], [9], [11], [16], [17], [18], these are not sufficiently comprehensive to regard γ-H2AX as established and validated for use as a biodosimeter. For these reasons and in complement to these works, we investigate γ-H2AX measurement as a irradiation biodosimeter in non-human primates subjected to total-body irradiation (TBI) with a non-lethal to lethal dose range, doses that are realistic for an accident scenario that could lead to long-time recovery for DNA damage repair. We report that yield and distribution of γ-H2AX foci in lymphocytes taken from macaques subjected to TBI can be used to estimate radiation exposure at doses in the medical triage range for several days after irradiation. Furthermore, we show that plucked hair bulbs exhibit γ-H2AX foci in response to IR and may also be utilized to help evaluate radiation exposures.

We conclude that γ-H2AX biodosimetry is a robust tool for estimating radiation exposure, injury and dose following TBI in the non-lethal to lethal dose range utilizing lymphocytes and plucked hairs taken from subjects up to several days after exposure.

## Results

### γ-H2AX detection in macaque and human lymphocytes *ex vivo*

**Figure 1:**
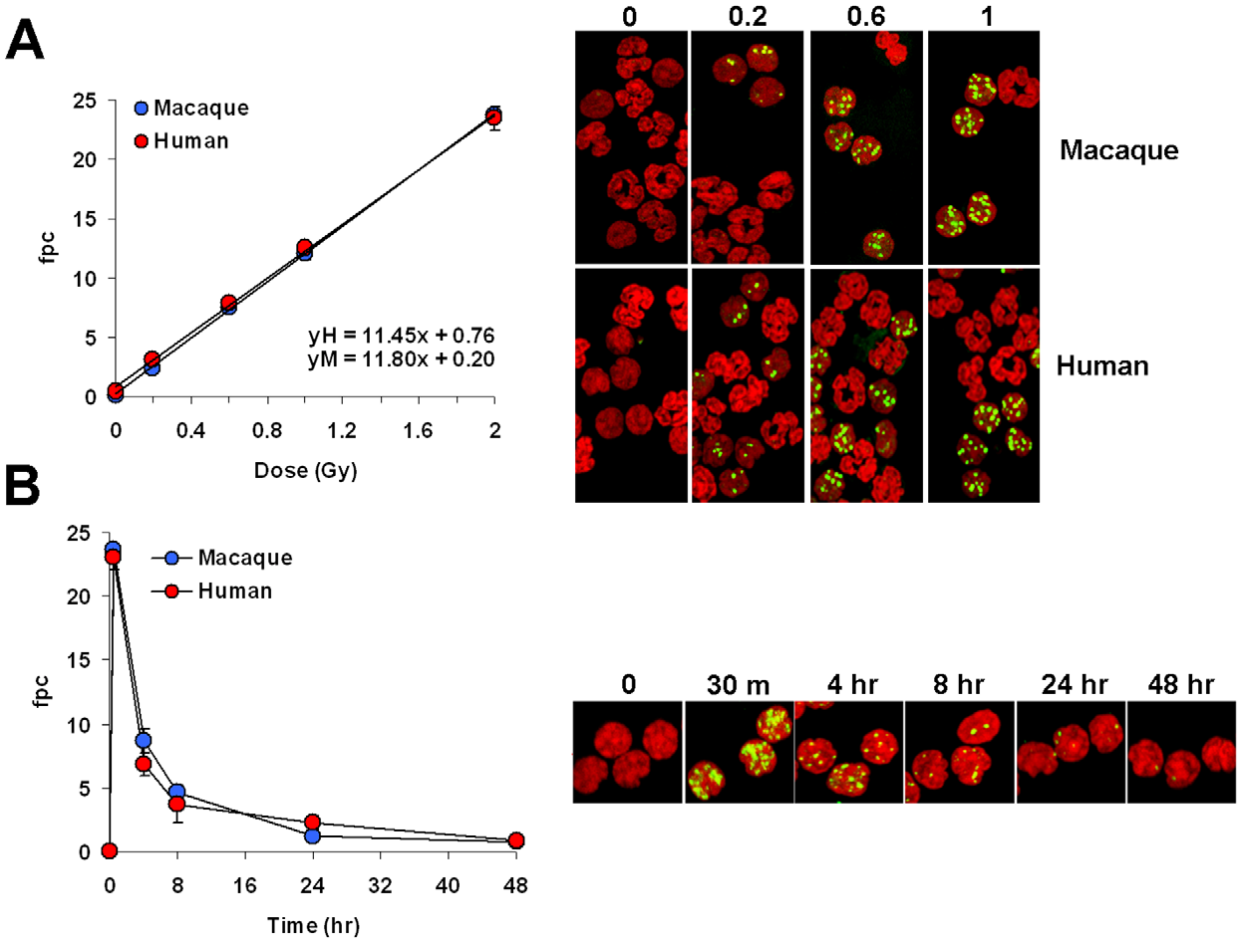
γ-H2AX foci in macaque and human peripheral blood white cells exposed to IR *ex vivo*. (**A**) *Left panel*, Incidence of γ-H2AX foci in human and macaque blood samples irradiated *ex vivo*. Blood samples were irradiated with zero, 0.2, 0.6, 1 and 2 Gy, incubated at 37°C for 30 min, then processed for γ-H2AX foci counting. Values are average numbers of γ-H2AX foci per cell (fpc). Error bars indicate standard deviations (n = 3). *Right panel*, Representative images of white cell preparations used for fpc determinations shown in the left panel. Green, γ-H2AX; red, DNA stained with PI. Arrowheads denote some of the polymorphonuclear cells. (**B**) *Left panel*, Incidence of γ-H2AX foci in macaque and human lymphocytes at various times after exposure to 2 Gy. Experimental procedures were as described above. Data presented as averages ± standard deviations (n = 3). *Right panel*, Representative images of white cell preparations used for the fpc determinations shown in the left panel. Green, γ-H2AX; red, DNA stained with propidium iodide (PI).

To evaluate the appropriateness of the macaque model for γ-H2AX biodosimeter studies in humans, we compared the kinetics of γ-H2AX foci incidence in macaque and human lymphocytes. Whole-blood samples were irradiated *ex vivo* with zero to 2 Gy and fixed 30 min to 48 hours later (Fig. 1). White cells were spotted on slides and stained for γ-H2AX. We restricted our γ-H2AX foci measurements to lymphocytes with spherical nuclei, because while lymphocytes generated a robust γ-H2AX response to these doses of irradiation, no substantial γ-H2AX signals were visible in the polymorphonuclear cells (Fig. 1A, right panel).

Like their human counterparts, macaque lymphocytes were found to respond robustly to irradiation over the range of 0.2 to 2 Gy, exhibiting a proportional relationship between the number of γ-H2AX foci and irradiation dose that was indistinguishable from that of the human lymphocytes (Fig. 1A). In addition, the rates of γ-H2AX foci disappearance in macaque and human lymphocytes up to 48 hours after exposure to 2 Gy were indistinguishable (Fig. 1B). However, γ-H2AX foci were still present 48-hours post exposure in both macaque and human lymphocytes at about 3% of the maximal level. While this value may seem low, it is still generally well above the control levels (0.75±0.04 at 48 hours *vs.* 0.03±0.01 in macaques). The virtually identical behavior of human and macaque lymphocytes indicates that the macaque makes an excellent model for developing γ-H2AX foci quantification as a biodosimeter for human radiation exposure.

### γ-H2AX foci in lymphocytes after TBI of macaques

**Figure 2:**
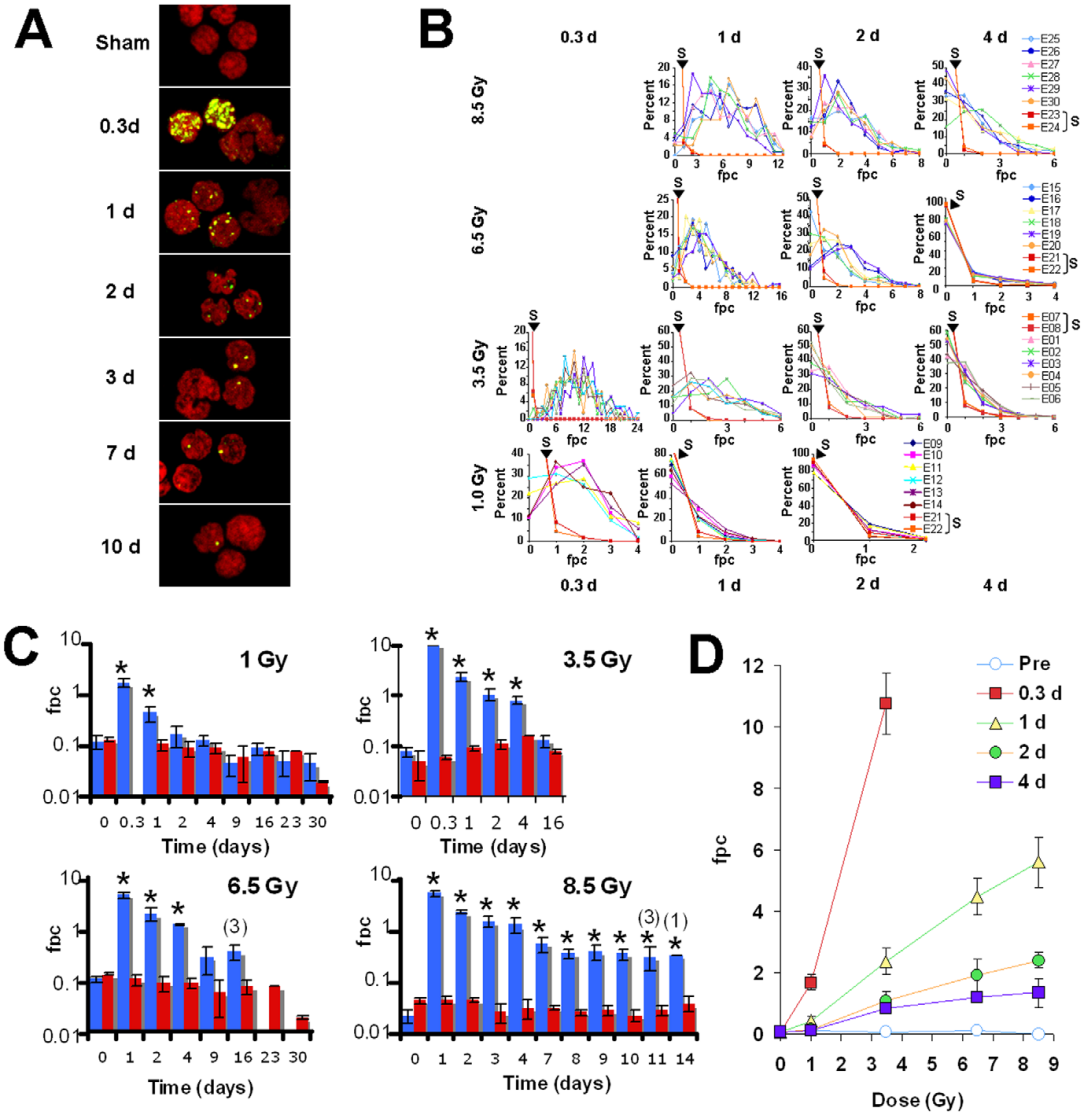
Kinetics for γ-H2AX foci loss in macaque lymphocytes after total body irradiation. (**A**) Representative images of macaque lymphocytes in Sham-irradiated (Sham) and 0.3, 1, 2, 3, 7 and 10 days (d) after 8.5 Gy-TBI. As previously observed with blood samples irradiated *ex-vivo*, the γ-H2AX foci were fewer and fainter in neutrophils than in lymphocytes. Green, γ-H2AX; red, DNA stained with PI. Arrowheads indicate neutrophils. (**B**) γ-H2AX foci distribution in lymphocytes taken from individual animals subjected to TBI. The rows from top to bottom show the focal distributions from individual animals after 8.5 Gy-TBI (6 macaques E25 to E30) and Sham-TBI (2 macaques E23 and E24, noted S), 6.5 Gy-TBI (6 macaques E15 toE20) and Sham-TBI (2 macaques E21 and E22, noted S), 3.5 Gy-TBI (6 macaques E01to E06) and Sham-TBI (2 macaques E07and E08, noted S), and 1 Gy-TBI (6 macaques E09 to E14) and sham-TBI (2 macaques E21 and E22, noted S). Results from the sham-IR are shown with red lines (noted S). The columns from left to right correspond to the different sampling times post-TBI, 0.3 d (1 and 3.5 Gy), 1 d (1 , 3.5 , 6.5 and 8.5 Gy), 2 d (1 , 3.5 , 6.5 and 8.5 Gy), and 4 d (3.5 , 6.5 and 8.5 Gy). (**C**) Values for γ-H2AX foci per cell *vs* days after the indicated TBI doses. The data for the TBI treated cohorts (blue bars) and sham treated controls for each cohort (red bars) are plotted on a log scale to bring out the details concerning differences between the TBI and sham treated animals. The data are shown as mean numbers of γ-H2AX foci per lymphocyte from each cohort ± standard deviations (n = 2 for Sham irradiated samples; n = 6 for TBI-irradiation, except 16 days after 3.5 Gy, n = 3 and 11 days after 8.5 Gy; no SD was determined 14 days after 8.5 Gy as data were prepared from 1 animal). (**D**) Foci per cell values in lymphocytes taken at the noted time (Pre = the day before TBI) and plotted *vs* TBI dose. Data are means ± standard deviations (n = 6). Equations for trendlines and R-squared values are y = −0.005x+0.096 / R^2^ = 0.225 for Pre-IR; y = 3.167x−0.578 / R^2^ = 0.980 for 0.3 d; y = 0.672x−0.034 / R^2^ = 0.996 for 1 d; y = 0.286x+0.014 / R^2^ = 0.993 for 2 d and y = 0.158x+0.097 / R^2^ = 0.956 for 4 d. Asterisks in (**C**) denote statistically difference between TBI-and sham-irradiated macaques (P<0.05, t-test). Detailed statistical analysis is shown in **Table 2**. Numbers in (**C**) for the 6.5 and 8.5 Gy panels correspond to the numbers of animals alive after TBI at the indicated time (no number indicates that all 6 TBI-irradiated animals are alive).

Individual macaques were subjected to TBI with a ^60^Co source at a rate of ∼0.6 Gy per minute. Thirty individuals were placed in 5 groups, each receiving a different TBI dose of 0 (sham), 1, 3.5, 6.5 and 8.5 Gy. These doses were chosen to permit an evaluation of multiple biodosimetric assays over a broad dose range. At times ranging from before radiation exposure to 23 days after TBI, blood was drawn and separated into plasma and buffy coat fractions. Upon receipt, all buffy coat fractions were processed for γ-H2AX detection and 100–200 lymphocytes from each sample were used for γ-H2AX quantitation (Fig 2A).

Figure 2B shows the distribution graphs of γ-H2AX foci in the lymphocyte populations of different individual animals. These graphs of animals of the same cohort at the same time point overlay each other, indicating that the different animals responded similarly to TBI. However, their responses are not identical, giving rise to the SD values shown in Figures 2C and 2D. The distribution graphs of γ-H2AX foci in the two sham-treated animals in each cohort are also shown, revealing lymphocyte populations containing a few percent with one focus, and rarely a few cells with two foci (Fig. 2B, red lines).

The mean values of foci per cell (fpc) for Sham-irradiated controls and TBI are listed in Tables 1 and 2 respectively and are plotted *vs* time after exposure (Fig. 2C) and *vs* irradiation dose (Fig. 2D). Values for 1-Gy exposure are significantly different than the corresponding values from the sham-irradiated controls at 0.3 and 1 day post exposure (Fig. 2C and 2D, 1.69±0.25 and 0.43±0.14 respectively *vs* 0.10±0.02; p<0.05), but at 2 days post exposure, the fpc values had returned almost to control levels (Fig. 2C and 2D, 0.17±0.07 *vs* 0.08±0.02; p = 0.218).

**Table 1:** Values for γ-H2AX foci per cell in macaque lymphocytes for sham-irradiation at the indicated times in days (d).

	Sham (1 Gy)Mean ± SD	Sham (3.5 Gy)Mean ± SD	Sham (6.5 Gy)Mean ± SD	Sham (8.5 Gy)Mean ± SD
Pre-IR	0.131±0.007	0.051±0.031	0.131±0.07	0.043±0.006
0.3 d		0.060±0.006		0.043±0.006
1 d	0.104±0.028	0.089±o.o10	0.104±0.028	
2 d	0.088±0.028	0.110±0.024	0.088±0.028	0.045±0.009
3 d				0.044±0.004
4 d	0.089±0.019	0.166±0.001	0.089±0.019	0.026±0.010
7 d				0.032±0.013
8 d				0.031±0.003
9 d	0.056±0.038		0.056±0.038	0.026±0.003
10 d				0.027±0.005
11 d				0.022±0.005
14 d				0.028±0.006
16 d23 d	0.074±0.0120.074±0.001	0.078±0.006	0.074±0.0200.074±0.001	0.038±0.013

Blanks indicate no data for the corresponding times. Pre-IR: values for γ-H2AX foci per cell prior to irradiation.

**Table 2:** Values for γ-H2AX foci per cell in macaque lymphocytes after total body irradiation with doses of 1, 3.5, 6.5 and 8,5 Gy at the indicated times in days (d).

	1 GyMean ± SD	3.5 GyMean ± SD	6.5 GyMean ± SD	8.5 GyMean ± SD
Pre-IR	0.118±0.034	0.078±0.017	0.104±0.011	0.021±0.006
0.3 d	1.696±0.251	10.754±0.995(***)		
1 d	0.438±0.146(*)	2.364±0.431(***)	4.424±0.567(***)	5.610±0.842(***)
2 d	0.170±0.078	1.084±0.302(**)	1.909±0.535(**)	2.406±0.265(***)
3 d				1.541±0.356(**)
4 d	0.126±0.028	0.829±0.157(**)	1.192±0.066(***)	1.347±0.464(**)
7 d				0.561±0.183(**)
8 d				0.365±0.078(**)
9 d	0.044±0.021		0.274±0.142	0.394±0.132(*)
10 d				0.365±0.099(**)
11 d				0.322±0.156
14 d				0.350(*)
16 d23 d	0.087±0.0220.049±0.024	0.131±0.035	0.365±0.119	

Blanks indicate no data for the corresponding times and doses. Pre-IR: values for γ-H2AX foci per cell prior to irradiation. Asterisks denote statistically difference between TBI-and sham-irradiated macaques ((*): P<0.05, (**): P<0.01, (***): P<0.001; t-test).

With both 3.5- and 6.5 Gy-TBI, γ-H2AX foci distribution differed significantly from those of the sham-treated animals up to 4 days after exposure (Fig. 2C and 2D). The fpc values at 4 days post exposure were 0.82±0.15 after 3.5 Gy-TBI and 1.19±0.06 after 6.5 Gy-TBI, about five (±1)-fold and 15 (±2)-fold above the corresponding control values of 0.16±0.00 and 0.08±0.01 (Fig. 2C and 2D and Tables 1 and 2). The subsequent sample was obtained at 16 days post-irradiation for 3.5 Gy-TBI and its value was statistically indistinguishable from the control value (Tables 1 and 2 and Fig. 2C). For 6.5 Gy, subsequent samples were obtained 9 and 16 days post-irradiation, and while the fpc values remained above the control values at these time points, the difference at 9 days post-irradiation was below the level of significance (Fig. 2C and Tables 1 and 2).

For 8.5 Gy-TBI, residual foci were observed after each sample withdrawal for up to 14 days with all but 1 fpc value being statistically different from those of the corresponding sham-irradiated controls (Fig. 2C and Tables 1 and 2; p<0.001 for days 1 and 2 post-IR; p<0.01 for days 3, 4, 7, 8 and 10 post-IR; p<0.05 for day 9 post-IR and 14 post-IR, ; p = 0.053 for day 11 post-IR). The fpc values at 4 days were 1.34±0.46, well above the control values of 0.02±0.01 and at 9, 10, 11 and 14 days post-irradiation the values in the surviving animals were 0.39 ±0.13, 0.36 ±0.09, 0.32 ±0.15 and 0.35 ±0.0 respectively *vs* 0.02±0.00 for the sham-irradiated control animals. The two doses of 6.5 and 8.5 Gy were lethal to all the animals, and in contrast to the lower doses, the fpc values remained elevated for these two doses at two weeks post exposure (Fig. 2C). Importantly, fpc values increase linearly with the irradiation dose (especially at doses greater than 1 Gy) and are substantially greater than the values for the sham-irradiation controls after 4 days (Fig. 2D).

### γ-H2AX in plucked hairs after TBI

**Figure 3:**
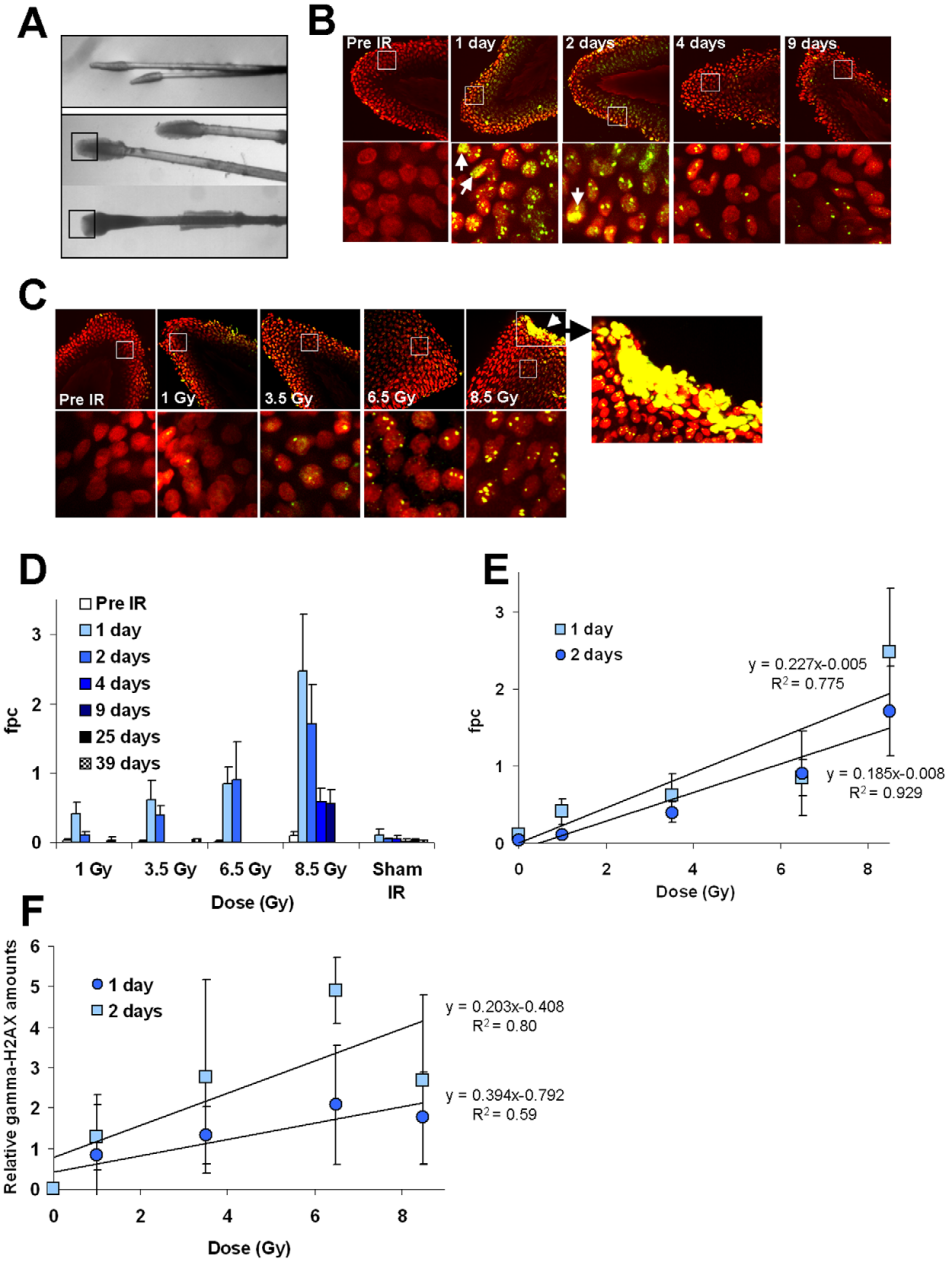
Kinetics for γ-H2AX foci in macaque plucked hairs after total-body irradiation. (**A**) Representatives images of plucked hairs obtained from the fur and the head (upper panel) and from eyebrows and whiskers obtained from the face (lower panels). Plucked eyebrow hairs and whiskers with morphologies similar to the ones shown in the lower panels were found suitable for γ-H2AX detection. The black squares in the lower panel indicate the region of interest for γ-H2AX microscopy. (**B and C**) Representative images of sampled hair bulb regions of eyebrow hairs and whiskers taken from NHPs at the indicated times after 8.5 Gy-TBI (**B**) and at 1 day after the indicated TBI doses (**C**) are shown. Images in the bottom row are enlargements of the regions inside the white squares in the upper images. The rightmost image in (**C**) is an enlargement of the apoptotic region in the adjacent image. Arrows denote apoptotic cells in the other images. Green, γ-H2AX; red, DNA stained with PI. (**D**) Kinetics for γ-H2AX foci loss in plucked hairs for pre-irradiation (Pre IR), sham-irradiation (Sham IR) and at time points after the indicated TBI dose. Images of the hair apex were taken using laser-scanning confocal microscopy and γ-H2AX foci were counted in 100–500 cells. fpc: foci per cell. (**E**) The incidence of γ-H2AX foci at 1 and 2 days post TBI obtained by counting foci. Error bars signify standard deviations (n≥3). fpc: foci per cell. Least squares regression analysis show a direct relationship between radiation doses and γ-H2AX signals (slopes = 0.260103 and 0.194473 and correlation coefficients r>0, r = 0.844 and 0.880 for 1 d and 2 d post-IR respectively (p<0.001)). (**F**) Incidence of γ-H2AX relative amounts at 1 and 2 days post TBI obtained by immunoblotting. Error bars signify standard deviations (n≥2). Relative amounts of γ-H2AX in each sample were determined by comparison with total H2AX.

Plucked hair bulbs retain many of the cells present in the intact follicle. These cells have been investigated as possible monitors of drug activity or diagnosis [19], [20], [21] and to assess radiation exposure [22], [23].When we examined macaque plucked hair bulbs for the presence of γ-H2AX foci after TBI, we found that hair bulbs plucked from the fur generally did not contain sufficient material for analysis (Fig. 3A, top), however, those from the eyebrows and whiskers (Fig. 3A, bottom) were amenable to γ-H2AX foci analysis. Hairs were plucked from animals, 1 day before, 1 and 2 days after 1, 3.5 and 6.5 Gy TBI and 1, 2, 3, 4 and 9 days after 8.5 Gy TBI. Plucked hairs taken from unirradiated (sham) animals contained few γ-H2AX foci (Figs. 3B and 3C, left most images), with values of 0.04±0.03 indicating that the act of plucking in itself does not significantly induces γ-H2AX foci formation under these assay conditions.

Figure 3B and 3C are representative images showing γ-H2AX foci in hairs plucked from unirradiated macaques and macaques after TBI. As previously observed with lymphocytes, the frequency of γ-H2AX residual foci in plucked hairs was dose dependent (Fig. 3E). However, in contrast to lymphocytes, the incidence of γ-H2AX foci on days 1 and 2 were quite similar at 3.5 Gy and above (3D and 3E). With hairs plucked after 8.5 Gy exposure, the highest γ-H2AX signal were observed 1 and 2 days post-irradiation (2.47±0.80 and 1.70±0.57), followed by similar decreases on days 4 and 9 post-irradiation (0.58±0.19 and 0.56±0.19) (Fig. 3D).

We also analyzed γ-H2AX levels relative to total H2AX levels by immunoblotting (Fig 3F). Analysis of plucked hairs by immunoblotting shows an increase of γ-H2AX levels in a dose dependent manner. Yet, the increase in γ-H2AX signals with doses, did not reach statistical difference. Surprisingly, the immunoblot results exhibited somewhat higher relative levels of γ-H2AX on day 2 compared to day 1. This may be explained at least in part by the accumulation of apoptotic cells in the hair bulbs following the larger TBI doses (Fig. 3C). Apoptotic cells contain very much larger amounts of γ-H2AX compared to that contained in a focus, thus a relatively small number of apoptotic cells could account for the increased γ-H2AX signal [4].

These results suggest that plucked hair bulbs may be a useful tissue for assessing exposures to ionizing radiation, either in conjunction with lymphocytes or by themselves.

## Discussion

Our results with non-human primates support the proof-of-concept that measurement of γ-H2AX foci in lymphocytes and plucked hair bulbs may be useful for estimation of both homogeneous and inhomogeneous exposures to irradiation at times at least 4-days post exposure at doses of 3.5 Gy and above. While γ-H2AX foci numbers decrease after reaching the maximum at 30-min post exposure with initial half-times of ∼2–3 h to levels ∼3–5% of the maximal value at 2 days, these values are still ∼10 times the control values [24] and this study). Beyond 2 days, the half-time of loss is much slower. At 3.5 Gy, the fpc values at 4 days are 70% those at 2 days. With both 6.5 and 8.5 Gy, the fpc values remained elevated at levels 5–10 fold above the control values until sacrifice about 2 weeks later, levels we estimate to be ∼0.4% of the initial fpc values. These observations suggest that a fraction of irradiation-induced DSBs remain unrepaired at these higher doses. There are, however, no definitive studies that have characterized the molecular nature of these residual foci. Such foci may result from the loss of genetic material with γ-H2AX signal being located at a chromosome broken end or may represent DNA lesions complexes, perhaps including clustered lesions. These structures may potentially be the source for the formation of radiation-induced chromosomal aberrations. Using quantitative immunofluorescence microscopy analysis, a dose response for γ-H2AX foci have also been reported in mouse skin at 30 minutes, 24 hours, and 7 days after whole-body irradiation with doses of 1 to 10 Gy [25]. The authors of this study conclude that γ-H2AX detection by microscopy may be a useful biodosimeter to determine dose at times up to 1 week after radiation exposure *in vivo*.

Plucking hairs is noninvasive and fairly painless compared to other types of sample collection. Since it could be performed by non-clinical personnel, it may be well suited for large scale sample collection. A recent study showed the feasibility of using γ-H2AX as a marker for DNA damage in plucked hairs from patients undergoing chemotherapy [26].

We show that the detection of γ-H2AX in plucked hairs can be used to monitor TBI for several days after exposure. Moreover, it is potentially advantageous that the γ-H2AX signal decreases little or not at all between 1 and 2 days post-irradiation. The slower rate of γ-H2AX foci loss in hair bulbs compared to lymphocytes may be due to a lack of repair in hair differentiating hair cells or to the inability for these cells to be removed, in contrast to the constant cleansing of the blood of severely damaged lymphocytes [27], [28] (Fig. 3B and 3C, arrows). This point is relevant since H2AX phosphorylation is present and in fact is dramatically elevated in apoptotic cells [29], [30].

Additional studies are required to evaluate the influence of potential confounders (i.e., inter-individual variations, dose fractionation, response of special populations (i.e., elderly, etc.) for use of this assay for radiation injury and dose assessment. Bhogal et al recently showed that γ-H2AX foci incidence in mouse skin exhibited substantial differences between inbred strains, with scid mice exhibiting the highest foci density [25]. However, mice of the same strain appeared to respond similarly. The similar irradiation response among macaques, which are not inbred, suggests that responses may be expected to be similar among members of large human populations.

In summary, measuring γ-H2AX levels in lymphocytes and plucked hair bulbs appears to be a promising assay for estimating the extent and homogeneity of radiation exposure even when samples are taken several days after exposure. This approach could be useful for several practical biodosimetry applications including the ability: a) to rapidly identify and triage severely exposed individuals in radiation accidents and radiological terrorism incidents, and b) to identify severely-exposed individuals (whole-body doses 3 to 8 Gy) and hence candidates for cytokine therapy [31].

## Materials and Methods

### Model systems and biosampling

Human peripheral blood sample from five healthy human donors (4 males, 1 female; ranging from 31 to 72 years old) collection was performed at the NIH blood bank in accordance with NIH regulations similar to that previously described [24]. Male and female rhesus monkeys (*Macaca mulatta*) (∼5.5 kg; ∼4-yr old at time of exposure; n = 28; two sham-treated animals were reused in a radiation cohort), were housed in individual stainless-steel cages in conventional holding rooms at the Armed Forces Radiobiology Research Institute's Veterinary Sciences Department in an animal facility accredited by the Association for Assessment and Accreditation of Laboratory Animal Care (AAALAC) International. Research with animals was conducted according to the principles enunciated in the Guide for the Care and Use of Laboratory Animals prepared by the Institute of Laboratory Animal Resources, National Research Council. Peripheral blood biosampling using the Macaque *ex vivo* radiation model system was performed similar to that previously described [32]. Peripheral blood (<1.5×10^−3^ l) was drawn from ketamine-anesthetized animals by saphenous vein into potassium EDTA (Cat.# 365974, Becton Dickinson) vacutainer tube at designated time prior- and post-irradiation. Blood samples were stored on ice for several hours until use.

Hair bulbs were collected from ketamine- (Ketaset® [10 mg kg^−1^, i.m.], Fort Dodge Laboratories; Fort Dodge, Indiana) anesthetized macaque rhesus monkeys by plucking individual hairs from eyebrow and whiskers. Hair bulbs with attached hair shaft were placed into 15-ml centrifuge tubes containing phosphate buffered saline (PBS) at maintained at ice-bath temperatures (∼4°C) until further processing for γ-H2AX immunocytochemistry.

### Radiation exposure and dosimetry

*Ex vivo* radiation exposures were performed similar to that previously described [24]. Human and macaque blood samples maintained on ice were exposed *ex vivo* to 0.2, 0.6, 1 and 2 Gy doses of ^137^Cs γ-rays (dose rate: 0.69 Gy min^−1^) in a Mark-1 γ-irradiator (JL Sherpherd & Associates, San Fernando, CA). Sham-irradiated and irradiated blood samples were treated the same way. Samples were incubated at 37°C for various designated post-exposure times.

*In vivo* radiation exposure and dosimetry of Rhesus macaques were performed similar to that previous described [32]. Briefly, rhesus monkeys received total-body exposure to midline tissue doses of 1.0, 3.5, 6.5 and 8.5 Gy (n = 6 per dose cohort), ^60^Co- γ irradiation at nominal dose rate of ∼60 cGy min^−1^. During irradiation of each group, 2 macaques were sham irradiated (except for the groups receiving 1 Gy and 6.5 Gy. These two cohorts were irradiated on the same day and the same pair of macaques served as the sham controls for both). The doses were selected as part of a dose-response study designed to evaluate various candidate biodosimetry assays. Ketamine-anesthetized animals were placed in a plexiglass restraint chair, allowed to regain consciousness and were irradiated bi-laterally. Dosimetry was performed using an alanine/electron paramagnetic resonance system, with calibration factors traceable to the National Institute of Standards and Technology and confirmed by an additional check against the national standard ^60^Co source of the UK National Physics Laboratory.

### Lymphocyte preparation for γ-H2AX immunocytochemistry

Human and macaque buffy coats and blood samples were stored on ice prior to processing for γ-H2AX detection. White blood cells, including lymphocytes, were prepared for γ-H2AX detection after red cells removal by hypotonic lysis using glycerol (Invitrogen, Eugene, OR, USA) (see below). Blood samples or buffy coat aliquots (90 µl and 90 to 360 µl respectively) were transferred in 1-ml microfuge tubes and paraformaldehyde (PFA) (20% stock solution) was added to 2% final concentration. Samples were mixed for 5 sec and incubated for 20 min at 20°C. Fixed buffy coat samples were washed twice with 1-ml PBS by centrifugation at 4,000 rpm for 2 min (Microfuge 22R centrifuge, Beckman Coulter). Pellets were resuspended in 100-µl PBS and 2 volumes of 60% glycerol solution [Glycerol 100% diluted with PBS pH 7.4] were added. Blood samples were not washed after fixation with PFA and 2 volumes of 60% glycerol solution were directly added to the blood-PFA solution. The final percentage of glycerol in the mixture is 40%. After vortexing 5–10 sec., the mixture was incubated at 20°C for 45 min and then stored at −20°C if not used immediately.

Aliquots of 50 µl of the cells-Glycerol mixture were placed in 1.5 ml microfuge tubes containing 1 ml of PBS. After 5-sec vortex to mix the solution, the tubes were centrifuged for 2 min at 4,000 rpm. After centrifugation, the supernatants were discarded and pellets were resuspended in 1-ml PBS. After mixing and another round of centrifugation, pellets were resuspended in 200 µl PBS and were spotted on slides by cytospin for 4 min at 800 rpm. Staining for γ-H2AX was performed as previously described [24] with some modifications. For immunofluorescence, PBS was replaced with PBS containing 0.5% tween-20 and 0.1% triton X-100 for blocking and antibody incubations ( = PBS-TT). After cytospin, slides were incubated in PBS for 15 min, transferred to pre-chilled ethanol 70%, and then stored at 4°C overnight. Slides were then washed in PBS for 15 min and blocked with 5% BSA (Sigma) in PBS-TT for 30min. After a 5-min wash in PBS, slides were incubated 2 hr with the mouse monoclonal anti-γ-H2AX antibody (Abcam) [dilution 500 in 1% BSA in PBS-TT] followed with a 1-hr incubation with a goat anti-mouse Alexa-488-conjugated IgG (Invitrogen, Eugene, OR, USA). After a 5-min wash and a 20-min incubation at 37°C with RNAse A 0.5 mg/ml in PBS, slides were mounted with mounting medium containing propidium iodide (Vectashield, Vector Laboratories, Inc., Burlingame, CA) and sealed with nail polish. Laser-scanning confocal microscopy was performed with a Nikon PCM 2000 (Nikon Inc, Augusta, GA). Optical sections (0.5 µm) through the thickness of the sample were imaged and combined in a maximum projection with Simple32 software (Compix Inc.) so that all of the visible foci and bands in a nucleus or mitotic figure were recorded. The foci were visually counted in 50–200 cells.

### Hairs samples preparation for Western blotting

Four plucked eyebrow and whisker hairs with intact hair bulbs from 28 macaques were evaluated for γ-H2AX. Plucked hairs were transferred in 1.5-ml tube with PBS and kept on ice until whole cell proteins were extracted by SDS sample buffer. Plucked hair was placed on clean glass plate and a bulb was cut off with a clean and sharp razor blade under a magnify lens. Bulb parts from all four hairs were collected in 1.5-ml tube with SDS sample buffer including beta-mercaptoethanol. Detection for both γ-H2AX and H2AX were performed as previously described [33].

### Hair samples preparation for γ-H2AX immunohistochemistry

One hundred twenty four plucked eyebrow and whisker hairs with intact hair bulbs from 28 macaques were evaluated for γ-H2AX signal. Plucked hairs were transferred in 1.5-ml tube then fixed with 2% PFA for 20 min at room temperature. After fixation, PFA was aspirated and samples were washed with 1-ml PBS. After 3 washes with PBS, pre-chilled 70% ethanol was added to each tube and samples were kept at 4°C for up to one week. Plucked hairs were, then, washed with PBS for 5 min and placed on slides with the side of the bulb placed inside hydrophobic circles made using a Pap pen (Electron Microscopy Sciences, Hatfield, PA) and containing PBS while the other side of the hair was fixed to the slide with a drop of nail polish. As soon as the nail polish dried, slides were transferred into a jar containing PBS for 10 min.

Staining for γ-H2AX and mounting processes were performed as described above but all antibody incubations and washing times were increased by 50%. Laser-scanning confocal microscopy was performed with a Nikon PCM 2000 (Nikon Inc, Augusta, GA). Seven optical sections (0.8 µm) through the thickness of the hair tissue were imaged and combined in a maximum projection and the γ-H2AX foci were visually counted in all visible nuclei of the bulbs.

### Statistical Analysis

All values in this study were expressed as mean ±1 SD unless otherwise noted. The significant differences between the groups were analyzed by a Student's t test and P value of <0.05 was considered significant.
